# The use of trail cameras to monitor species inhabiting artificial nest boxes

**DOI:** 10.1002/ece3.8550

**Published:** 2022-02-07

**Authors:** Adrian Surmacki, Paweł Podkowa

**Affiliations:** ^1^ Department of Avian Biology and Ecology Faculty of Biology Adam Mickiewicz University Poznań Poland

**Keywords:** camera traps, cavity‐nesting, timing of egg laying, timing of hatching, video

## Abstract

1. Artificial boxes are commonly used in studies of cavity‐dwelling animals of various taxa. One advantage of nest boxes is that cameras can be used to monitor animals inside the cavity, however, most cameras used today have to be built de novo or modified or are expensive.
2. Here, we describe a method for monitoring nest boxes using off‐the‐shelf models of trail cameras that can record photographs and videos in daylight and darkness (TCM; Trail Camera Method). The cameras can record sequentially within a given time lapse or an infra‐red motion sensor can be triggered by activity in the nest box.
3. Using TCM in a Great Tit (*Parus major*) nest box population, we studied the hourly pattern of the first egg laying and the first egg hatching. We found that Great Tits laid eggs within 2 h of the sunrise while the timing of hatching spanned the 24‐h day. Moreover, we found that the hour of hatching affects the nestlings’ mass on the 2nd day of life, but not on the 12th day of life.
4. Comparing to traditional nest box checks, TMC requires about 75% less time to obtain data on the timing of egg laying and hatching. Moreover, the hour estimation error was several orders of magnitude greater with the traditional method.
5. Our data demonstrate that commercially available trail cameras are an affordable and convenient method of monitoring artificial cavities. Trail cameras are small, standalone, weather‐proof devices with integrated powering, memory storage, lighting, and recording systems. They could be easily swapped between boxes or removed. After small modifications of the box, they could be used to monitor a wide variety of behaviors of many animal taxa.

## Graphical abstract

We describe a novel method for monitoring nest boxes using off‐the‐shelf models of trail cameras that can record photographs and videos in daylight and darkness. Using trail camera setup inside Great Tit (*Parus major*) nest boxes, we recorded hours of the first egg laid and hatching. Our data demonstrate that commercially available trail cameras could be an affordable and convenient method of monitoring a wide variety of behaviors of many animal taxa living in artificial boxes.

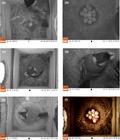

## 1 INTRODUCTION

Nest boxes are an important tool for conservation and scientific studies of cavity‐dwelling species including birds, mammals (e.g., Ciechanowski, 2005), reptiles, amphibians (e.g., McComb & Noble, 1981), and insects (e.g., Hilszczański et al., 2014) living in various climate zones (reviewed by Zárybnická et al., 2016). There are many advantages of using artificial cavities in scientific research. Because natural cavities are often limited, nest boxes can augment population sizes of rare species and allow researchers easy access to nesting cavities of rare and common species. Further, by slightly modifying boxes, researchers can monitor or manipulate environmental factors, for example, light (Podkowa & Surmacki, 2017), temperature (Bryan & Bryant, 1999), parasite loads (Heeb et al., 1998), or predation rates (Kaliński et al., 2014). As a consequence, cavity‐nesting species are often model species for studies of behavioral ecology, eco‐physiology, and genetics of free‐living animals (e.g. Delhey & Kempenaers, 2006; Lucas & Heeb, 2005; Roulin et al., 2007; Tschirren et al., 2005). However, nest boxes often provide substantially different conditions than natural cavities (e.g. Lambrechts et al., 2010; Maziarz et al., 2017; Wesołowski, 2011), therefore any conclusions derived from nest box studies should be raised with a special caution (Wesołowski, 2011).

Although studying box‐nesting species has its advantages, any attempt to study wild populations requires much time and effort because the standard set‐up consists of monitoring tens or even hundreds of nest boxes throughout the breeding season (e.g. Cole et al., 2012; Cresswell & McCleery, 2003; Glądalski et al., 2018). In bird studies, collecting data on the timing of nest building, egg laying, hatching, and fledging requires that researchers frequently monitor nests (summarized in Wang & Beissinger, 2011). Technical solutions have been developed to reduce field time (reviewed by Smith et al., 2015); photo and video technologies provide direct information about bird behavior inside nest boxes (e.g., Bambini et al., 2018; Hereward et al., 2021; Prinz et al., 2016; Zárybnická et al., 2016). Contemporary photography and real‐time recording systems have features appropriate for cavities, like miniature size, infrared sensitivity, movement‐induced trigger (Hereward et al., 2021; Prinz et al., 2016; Zárybnická et al., 2016). Despite these advantages, relatively few studies employ camera systems and often have small sample sizes, rarely exceeding 10 boxes (Haftorn, 1996; Cooper et al., 2009; Wang & Weathers, 2009; Ospina et al., 2015; Zárybnická et al., 2016, but see Bambini et al., 2018 for the exception). This is likely because there are not any ready‐to‐use commercially available nest box monitoring systems dedicated to scientific purposes. Indeed, many manufacturers offer special nest boxes integrated with small cameras (reviewed in Prinz et al., 2016). However, such solutions have many technical limitations and are suitable for monitoring a single garden nest box rather than large‐scale field research (Prinz et al., 2016). On the other hand, setting up nest‐monitoring systems requires electronic engineering and programming skills necessary to assemble and configure all components needed (e.g., lighting, cameras, data storage, triggering system, see Hereward et al., 2021; Prinz et al., 2016; Zárybnická et al., 2016). An additional problem with user‐built and commercial systems is their relatively high cost (Prinz et al., 2016; Zárybnická et al., 2016) as proper experimental design usually requires monitoring >100 nests.

Trail cameras (also called camera traps or game cameras) are one of the most promising types of cameras for monitoring animal behavior inside nest boxes. Trail cameras were originally used to monitor large, rare, and elusive mammals but subsequently have been used to study a diversity of taxa (Hobbs & Brehme, 2017; O’Brien & Kinnaird, 2008; Tremlett et al., 2020; Welbourne et al., 2019). When used for monitoring bird nests, camera trap studies have generally focused on feeding behavior of birds of prey and other large species (e.g., García‐Salgado et al., 2015; Harrison et al., 2019; López‐López & Urios, 2010). However, slightly modified trail cameras have the potential to monitor cup nests of small passerines (Uhe et al., 2020). Camera traps are also frequently used for the identification of nest predators and for the assessment of their behavior at birds’ nests (e.g., Ekanayake et al., 2015; Maziarz et al., 2017). In the past, trail cameras have only recorded bird activity outside of the cavity entrance (e.g., Griffiths et al., 2020). To our knowledge, trail cameras have never been used to monitor the interior of natural or artificial cavities of any animal species.

Here, we present a novel method of nest box monitoring using an off‐the‐shelf trail camera model (Trail Camera Method, hereafter TCM). The main goal of our study is to describe the details of the setup and discuss the pros and cons of using a trail camera to study selected aspects of the breeding biology of a cavity‐nesting bird, the Great Tit (*Parus major*). To show an example of the application of our method, we collected information about the time of day of egg laying and hatching. This is the first time when an ample sample of such a data was comprehensively gathered for one bird species. Based on theoretical calculations, we compared the effort and time expenditures of TCM to the traditional nest box checks. Finally, we presented methodological implications of using such data in studies on birds’ breeding biology and discussed possible applications of TCM in studies on animals’ behavior.

## 2 MATERIALS AND METHODS

### 2.1 Trail camera

**FIGURE 1:**
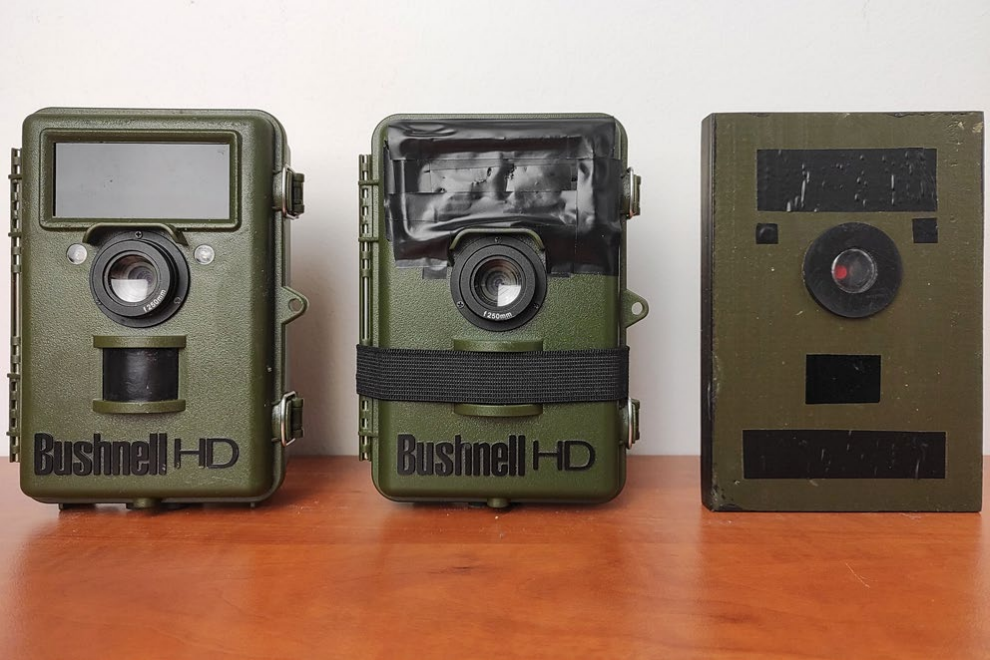
Bushnell Natureview^®^ HD trail camera used for nest box monitoring. From the left: trail camera with 250 mm lens without custom modifications, trail camera with IR LED lights covered with a black plastic wrap and PIR motion sensor covered with opaque rubber band, dummy trail camera

We used Bushnell Natureview^®^ HD trail cameras fitted with a 250 mm lens that enabled focusing at a minimal distance of 25 cm (Figure 1). The camera recorded photos (3–5 MP) in “Camera” mode, video clips (360, 640, 1080, 1920 MP) in “Video” mode, or both in “Hybrid” mode. It was triggered (within ca. 1 s) by moving objects with temperatures higher than the surrounding area which were detected by a Passive Infra‐Red motion sensor (PIR). Alternatively, photos or videos could have been recorded sequentially in even time intervals (1**–**60 min) within a given time lapse (“Field Scan” mode). Because it was not possible to turn off taking pictures triggered by the PIR sensor while using the “Field Scan” mode, we covered it with black opaque rubber bands (Figure 1). The device worked both in daylight and darkness due to IR LED flash, which turns on automatically when needed. During preliminary tests, we discovered that photos taken inside nest boxes were overexposed, even with minimal IR LED flash intensity (LED Control set to “Low”), which probably resulted from the short distance between the camera and the nest. To fix this problem, we covered IR LED flash with double layers of semi‐transparent black plastic wrap (Figure 1). During nest box monitoring, we set the PIR Sensor Level to a “High” position, which means that it was sensitive enough to detect even small differences in temperature between the bird’s body and the surrounding area. In “Camera” mode, the Night Vision shutter speed was set to “High” because most photos were taken in darkness and shorter exposition freezes motion better at the expense of brightness. All devices were powered by 12 AA Energizer^®^ Ultimate Lithium batteries. The overall dimensions of the trail camera were 155 × 115 × 60 mm and the weight was 505 g. We used 32 GB or 4 GB SD memory cards to store trail camera recordings. Because this model of trail camera had no built‐in monitor, we used a point‐and‐shoot camera to preview recordings from memory cards. For details of trail camera settings and specifications, see Appendix S1.

Before the breeding season, we placed dummy trail cameras in the place of real devices (Figure 1) to allow birds to acclimate. The sudden appearance of artificial objects, especially containing round, glossy elements (like a lens), may disturb brooding behavior (Heeb et al., 1998; Zárybnická et al., 2016). Dummy cameras were made of a wooden bar painted green and attached plastic elements mimicking a flash, PIR sensor, and the lens (Figure 1). We replaced dummies with trail cameras at advanced stages of nest building.

### 2.2 Nest boxes

**FIGURE 2:**
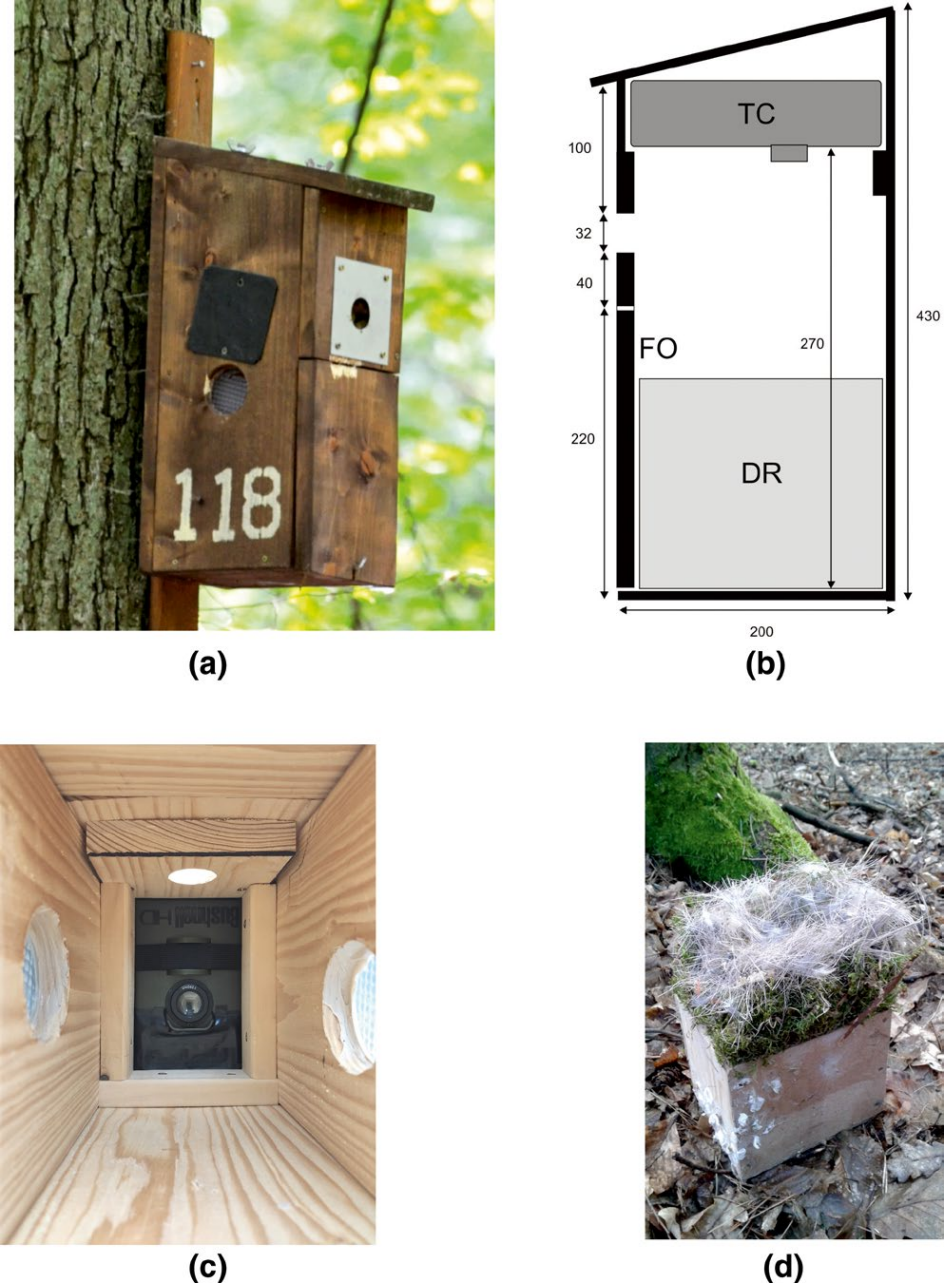
(a) Nest box in the field. (b) Design of the nest box equipped with the trail camera. (DR) nesting area in a drawer; (TC) trail camera in the chamber under removable roof; (FO) front opening. (c) Trail camera viewed from the nest box floor. (d) “Drawer” with a Great Tit nest

We constructed nest boxes using 2‐cm thick pine boards (inner dimensions: 40 × 16 × 12 cm, entrance hole diameter: 3.3 cm, entrance hole location: 24 cm above the floor, Figure 2). In the top part of the nest box, we built a chamber (5–8 × 16 × 12 cm), that opened downward in which we placed the trail camera (Figure 2). Access to the chamber was possible through a removable roof (Figure 2). The distance between the lens and the nest box floor was 30 cm. Trail cameras were located in the nest box horizontally, with the PIR sensor closer to the entrance (Figure 2). Access to the nests was assured by an opening (15 × 22 cm) in the lower part of the front wall (Figure 2). To facilitate access to eggs and nestlings, we built boxes with 10 × 10 × 12 cm “drawers”, made of two wood bars and three pieces of thin beaver wood on which birds built nests (Figure 2). To process eggs or nestlings, the drawer with a nest was removed from the nest box. Because of a concurrent study of nest illumination (Podkowa et al., 2019; Podkowa & Surmacki, 2017), nest boxes were fitted with two 5.0‐cm‐diameter resin windows 15 cm above the floor located in the side walls, which were equipped with an adjustable shutter made of 9 × 9 cm black plastic sheets (Figure 2). In nest boxes where windows were open, internal illumination was about 50 times higher compared to nest boxes with windows shut and in which the only source of light was the entrance hole (Podkowa & Surmacki, 2017).

### 2.3 Field methods

We conducted the study during the 2016 and 2017 breeding seasons in three study plots within Wielkopolski National Park in western Poland. Nest boxes (*n* = 159) were hung in 2014 at the height of about 3 m with entrances oriented southeast. Deciduous and mixed forests dominated the area (for more details of the study site see Podkowa & Surmacki, 2017 and Kudelska et al., 2017). To collect basic breeding biology data, we monitored all nest boxes every 2–5 days from mid‐March until late June.

To determine the time of laying of the first egg, we put trail cameras into nest boxes that had a nest cup with a lining. The trail camera was set to 24‐h “Field Scan” mode and “Camera” mode with the “Interval” set to 1 or 5 min. Thus, the device took one 3 MP photo every 1 or 5 min. To determine egg hatching time, we placed a trail camera in the box ~10–12th day of incubation. We assumed that the incubation starts on the day of laying last but one egg (Cramp & Perrins, 1993). The trail camera was set to 24‐h “Field Scan” mode and “Camera” mode with the “Interval” set to 1 min. The time of the first egg laid and the first egg hatched were read from the time‐stamp‐recorded file metadata (Supplementary Materials). The hatching day was the “0” day of the first nestling in the brood. Nestlings were weighted to the nearest 0.1 g using a digital balance on the 2nd and 12th day of their life.

### 2.4 The hour of the first egg

We used two methods to express the hour of the first egg laid. First (hereafter, “uncorrected timing of the first egg”), we used the hour of the first photo in which the egg appeared and applied this approach to all clutches (*n* = 56). Second (hereafter “corrected timing of the first egg”), the hour was delineated as the midpoint between the time of the last photo showing an empty nest and the first photo with an egg. We used the latter approach for 18 clutches in which both photos were less than one hour apart (5–50 min; mean ± SD = 29 ± 11 min). In remaining nests (*n* = 38), this approach was not feasible, because the photo of an empty nest was taken in the evening preceding the following morning when the first egg was photographed. During the time between these two photos (9–11 h), the content of the nest was constantly covered by a resting female. We compared both methods using the subsample of 18 nests for which the uncorrected hour of the first egg was calculated. The timing of laid eggs was expressed as minutes before/after the sunrise.

Great Tits often bury eggs in nest material (Haftorn & Slagsvold, 1995; Loukola et al., 2013), which can cause researchers to miss the time of the first egg appearing. To test the reliability of TCM in egg detection, we compared it with the results of field controls. During the nest inspection, the nest material was gently parted with the fingers to find eggs buried within it. In total, we analyzed data from 66 nest (29 in 2016; 37 in 2017).

### 2.5 The hour of the first egg hatched

**FIGURE 3:**
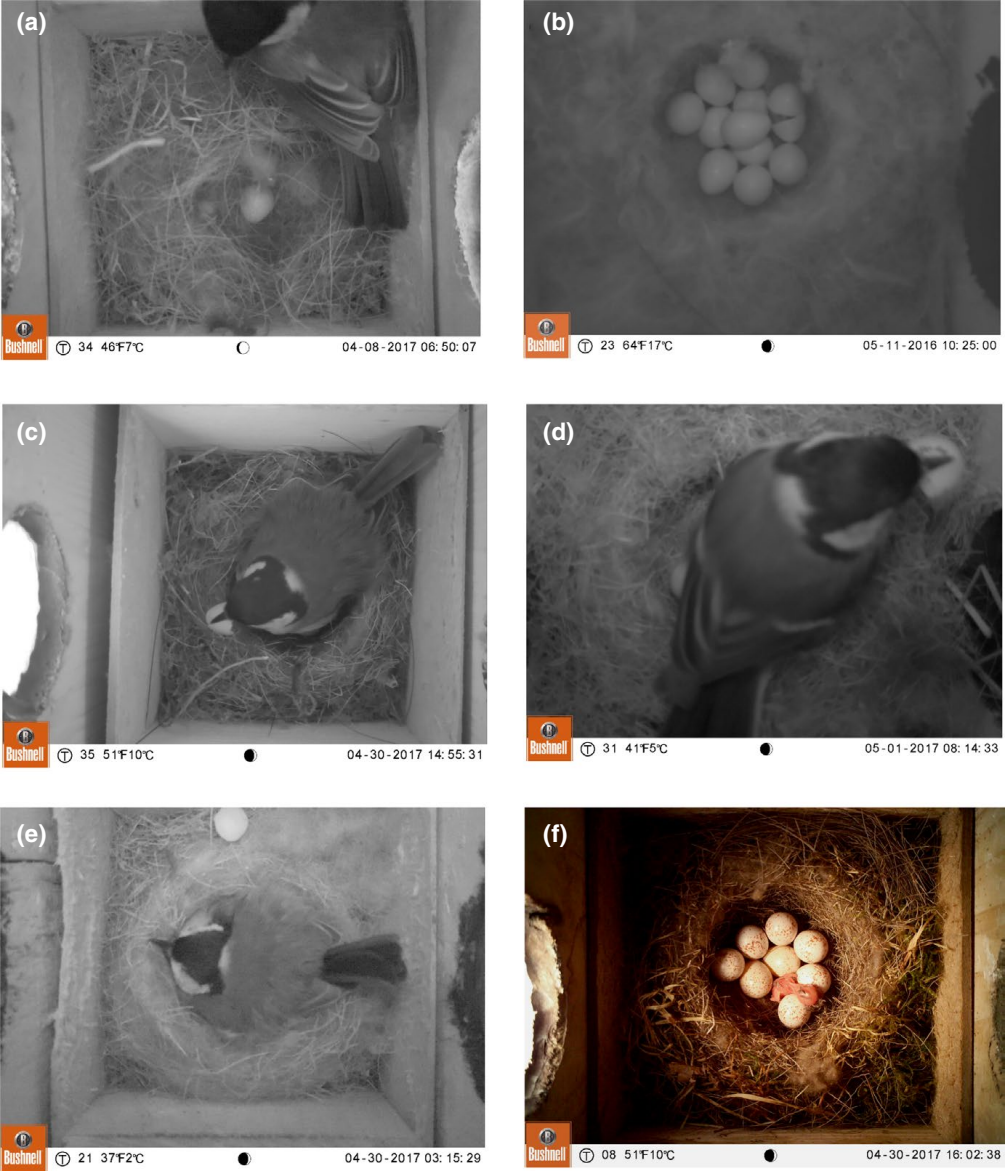
Samples of photos used in determining timing of egg laying and hatching: (a) the female with the egg laid; (b) hatching in progress; (c, d) female holding eggshell in a beak; (e) a piece of an eggshell removed from the nest; (f) freshly hatched nestling. Note that photos (b) and (d) were taken on high nests, therefore they are slightly out of focus

We established the hour that the first egg hatched for 42 nests in 2016 and 26 nests in 2017 (in total *n* = 68). Information about hatching hour was based on photos showing: (1) nestling during hatching process (*n* = 15), (2) freshly hatched nestling (*n* = 15), (3) pieces of eggshell on the edge of the nest or in female’s beak (*n* = 38, Figure 3). In all three cases, we assumed that the time of the egg hatching was the time at which the photo was recorded. We did so for three reasons. First, in nests where photos showed the first signs of hatching (cracks on the eggshell or emerging nestling), nestlings usually appeared in 5–10 min (own observations, Supplementary Materials). Second, freshly hatched nestlings have wet down feathers, suggesting they had recently emerged from the egg. Third, birds remove eggshells soon after hatching (Arnold, 1992; Tinbergen et al., 1962; Winkler, 2004, reviewed by Guigueno & Sealy, 2012). The timing of hatched eggs was expressed as minutes before/after the sunrise.

### 2.6 The assessment of efficiency and accuracy of TCM in relation to the traditional nest box checks

In the current study, we combined traditional nest box checks (hereafter NBC) with the TCM. We calculated the expected time effort needed to obtain the data on the hourly pattern of egg laying and hatching with the use of each method to assess their efficiency and accuracy. To date, there is no study in which NBC was used to survey hours of egg laying in Great Tit or any other tit species. The only study devoted to this topic was based on continuous video recordings (Haftorn, 1996). Therefore, to design NBC scenario, we used a modified protocol from the study on the timing of egg laying in European Starling (*Sturnus vulgaris*, Feare et al., 1982). We assumed that nest box checks started four days before the first egg was laid, what corresponds to the mean time between installing trail camera and the date of the first egg (mean ± SD = 3.6 ± 2.0). We assumed that each nest box was visited three times, between 5:00–7:00, 7:00–9:00, and 9:00–11:00 a.m. This corresponds to the time frame in which the eggs were laid according to TCM. Controls lasted until the egg was found. All unfinished nests (without the lining) were controlled every 3–4 days. In the TCM scenario, every 3–4 days, we controlled nest boxes with nests under construction to install or retrieve cameras.

To date, the hour of egg hatching was not studied in the Great Tit, nor any other cavity‐nesting bird species. Therefore, to design NBC scenario, we adopted the protocol from the study on egg hatching hours in open nesters (Skutch, 1952). According to it, nests were controlled three times during the day; dawn – noon, noon – afternoon, afternoon ‐ nightfall (Skutch, 1952). In NBC scenario, we assumed nest box controls in three two‐hour inspection intervals 5:00–7:00, 11:00–13:00, and 17:00–19:00. Controls lasted until the first egg was hatched. We assumed that nest box checks started two days before the first egg was hatched, what corresponds to the mean time between installing trail camera and the date of the egg hatching (mean ± SD = 2.3 ± 1.3). We did not consider more frequent controls nor night controls, due to a serious threat of brood desertion caused by researcher’s disturbances (Kania, 1989; Skutch, 1952). In the TCM scenario, all nest boxes were visited two days prior to the earliest expected day of the first hatch to install trail cameras. The second visit was performed in the middle of the egg‐hatching period. The third visit, during which cameras were retrieved, was performed after the last egg hatched.

In TCM and NBC scenarios for both egg laying and hatching, we assumed that the control of the single nest box lasts 10 min.

### 2.7 Statistical analysis

Because our data deviated from normal distribution (Shapiro‐Wilk test, *p* < .05 in all cases), we applied nonparametric tests accordingly (*U* Mann‐Whitney test, rank Spearman correlation). To investigate the effect of hatching time on nestling mass, we calculated the daytime (minutes) spent by the first hatched nestling throughout the day (hereafter, day‐time exposure). Daytime was the time between the sunrise and the sunset, which roughly corresponds to feeding hours in this population (Podkowa et al., 2019). Then we correlated it with mean nestling’s mass in the brood in the 2nd and 12th day of life (hatching day = 0). Analysis was performed for 39 broods measured in 2016.

## 3 RESULTS

Across nests, the number of photos needed to record the first egg ranged from 198 to 16,585 (mean: 2110,3) which used 29–8325 MB of card memory (mean: 1055,8 MB). The number of photos needed to record the first egg hatched ranged from 67 to 13,137 (mean: 2764) and used 36–6616 MB of card memory (mean 1433 MB). It means that one 32 GB or two‐three 4 GB SD cards could be used to record both egg laying and hatching in each nest. In a practice, for data safety, we used separate cards to record different stages of breeding. One set of batteries lasted to record both, the first egg laying and hatching.

By comparing data from TCM with field nest box checks, we found that trail camera detected the first egg in 85% of nests (*n* = 56). In 10 nests (15%), the first egg was not detected by the trail camera photos, while in two nests (3%) it was recorded by trail camera but missed during field nest checks.

The two measures of the timing of the first egg were significantly positively correlated (*r_s_* = .89, *p* < .01, *n* = 18). The “uncorrected timing” of the first egg was significantly later compared to the “corrected timing” (Wilcoxon rank test, *Z* = 3.73, *p* < .01); however, the difference was only ~17 min (median = 28.5 min, *Q*_25%–75%_ = 20–47; median = 11 min, *Q*_25%–75%_ = 6–27, respectively).

**FIGURE 4:**
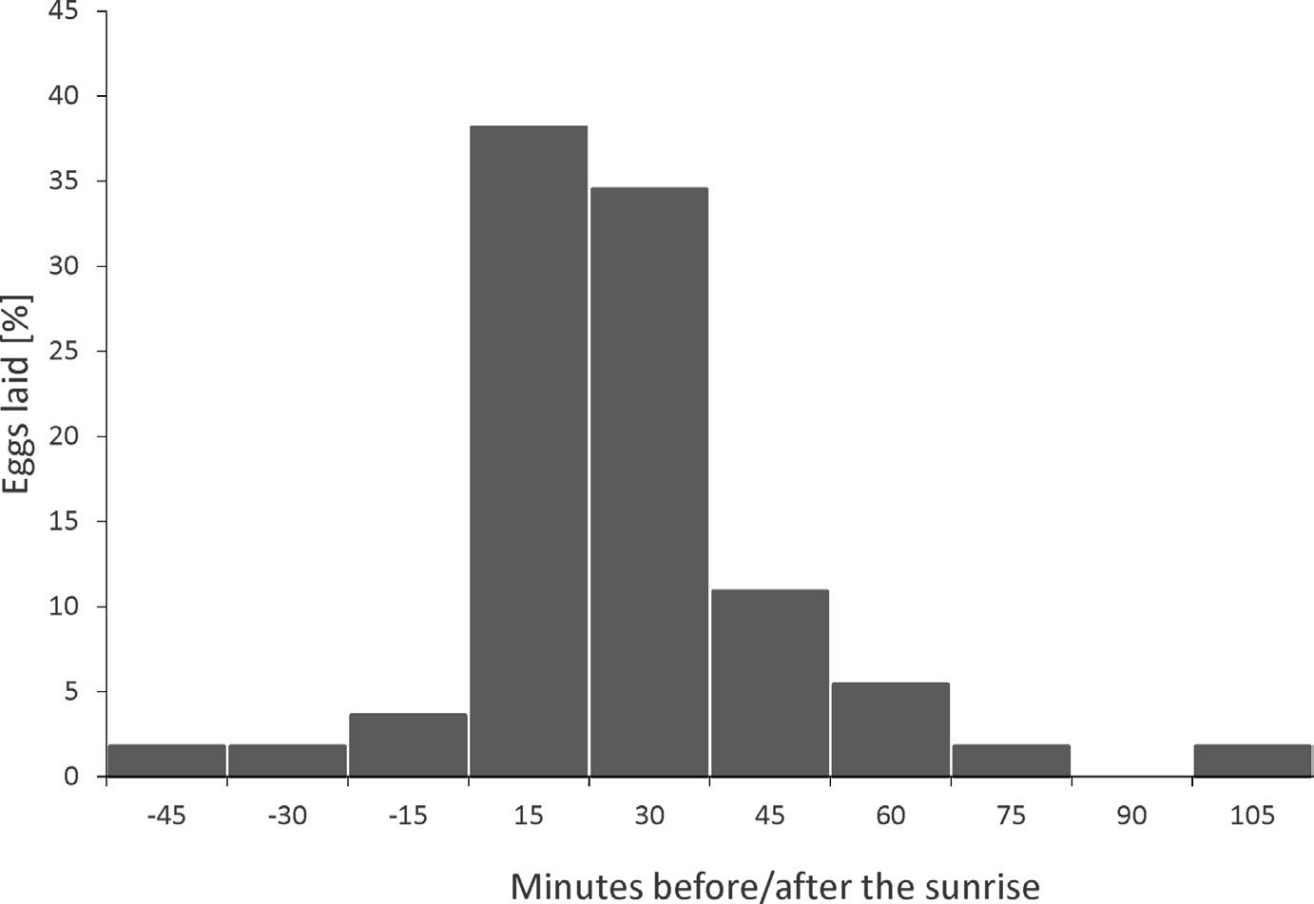
Distribution of the “uncorrected” laying hours of the first egg in relation to the sunrise (*n* = 55)

According to the “uncorrected timing”, in all but one case, females laid the first egg from 31 min before sunrise to 93 min after sunrise (Figure 4, median = 19, *Q*_25%–75%_ = 4.0, −6.5 to 11.0). The only exception occurred in one nest in 2017 when the egg was laid 274 min after sunrise. We did not detect a significant between‐year difference in the timing of egg laying (*U* Mann‐Whitney test, *Z* = −0.23, *p* = .82).

**FIGURE 5:**
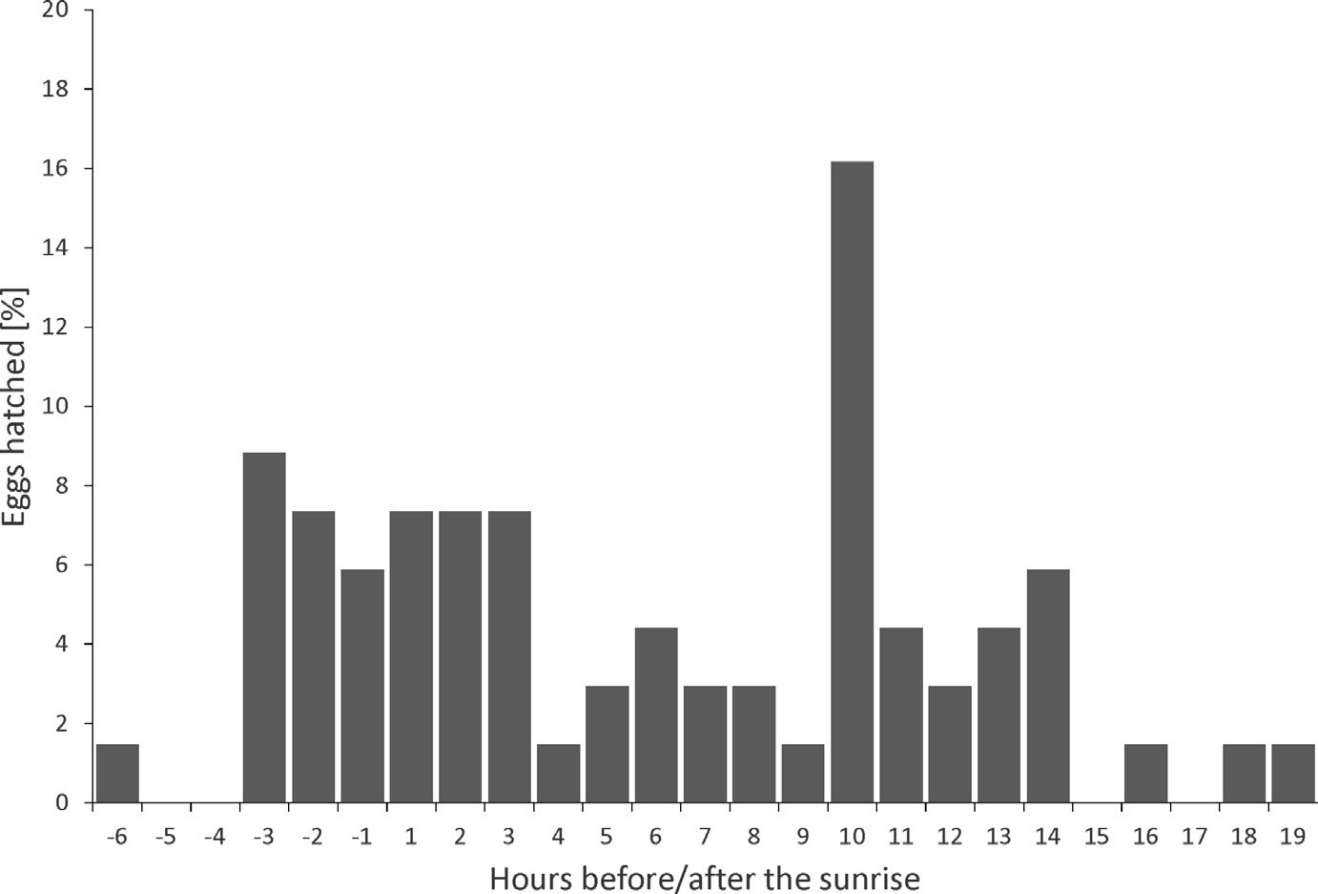
Distribution of hatching hours of the first eggs in relation to the sunrise (*n* = 68)

Great Tit eggs hatched throughout the 24‐h day (Figure 5). The earliest hatch was recorded at 5 h and 4 min before sunrise while the latest 18 h and 8 min after the sunrise. The median hour of hatching was 366 min after sunrise (*Q*_25%–75%_ = 60–623). About 44% of eggs hatched six hours around the sunrise (Figure 5). There was also a second smaller peak (16%) 10 h after sunrise (Figure 5). There was no difference in hatching time between 2016 and 2017 (*U* Mann‐Whitney test; *z* = −0.52, *p* = .60).

**FIGURE 6:**
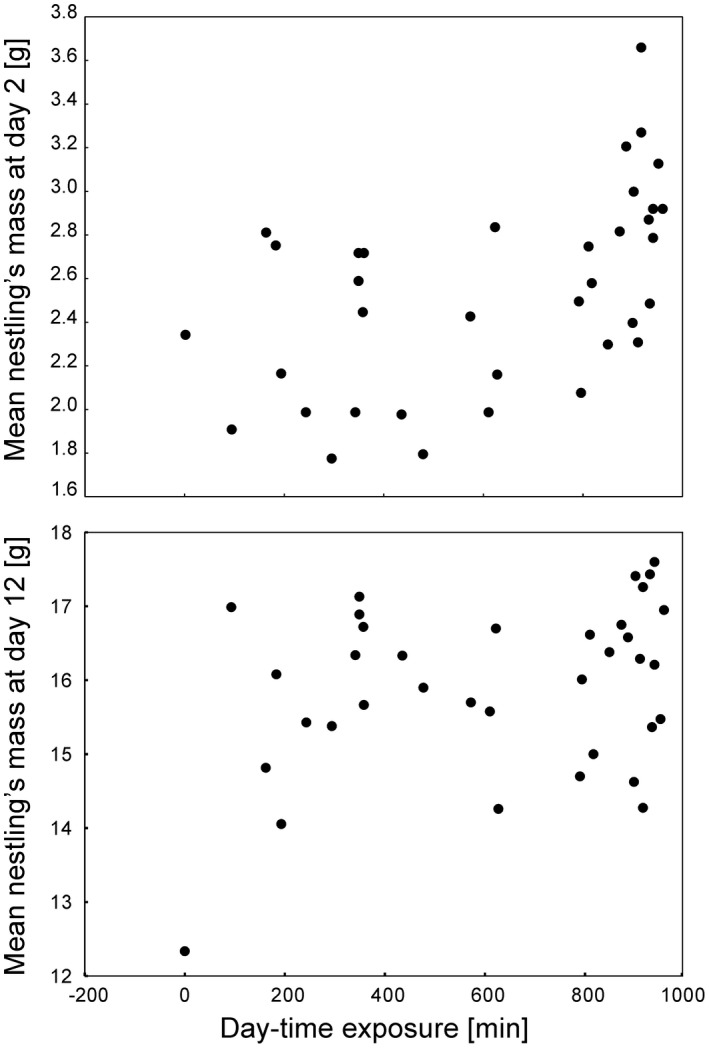
Relationships between mean nestling’s mass at the 2nd and 12th day of life and the exposure to a day‐time in the day of hatching. Graphs show data for 39 broods from 2016

Nestlings that spent more daytime in the day of their hatching were significantly heavier on the second day of life (*n* = 39, *r_s_* = .57, *p* < .001; Figure 6), but not on the 12th day of life (*n* = 39, *r_s_* = .27, *p* = .10; Figure 6).

Expected daily time expenditures needed to collect data on the hour egg laying and the hour of egg hatching did not differ between methods (*z* = 1.15, *p* = .25; *z* = −1.79, *p* = .07, respectively, Table 1). On the other hand, expected total time (minutes) spent on a field work was significantly lower for TCM than for NBC, both for hours of egg laying and hatching (χ^2^ = 168.09, df = 1, *p* < .01; χ^2^ = 224.23, df = 1, *p* < .01, respectively, Table 1).

**TABLE 1:** Estimation of the time effort needed to collect data on egg laying and hatching by means of Nest Box Checks and Trail Camera Method

	Nest Box Checks			Trail Camera Method		
Hour of egg laying	2016	2017	Total	2016	2017	Total
No. of field work days	18	15	33	5	5	10
Time per a workday (median, *Q*_25%_, *Q*_75%_)	275 (112.5, 300)	280 (150, 475)	280 (120, 360)	250 (150, 270)	270 (150, 290)	260 (150, 270)
No. of nest box controls	399	436	835	98	101	199
Total work time (min)	3990	4360	8350	980	1010	1990

Hour of egg hatching	2016	2017	Total	2016	2017	Total
No. of field work days	23	14	37	3	3	6
Time per a workday (median, *Q*_25%_, *Q*_75%_)	120 (30, 280)	150 (40, 210)	120 (30, 230)	420 (110, 420)	260 (80, 260)	250 (110, 420)
No. of nest box controls	337	217	554	95	60	155
Total work time (min)	3370	2170	5540	950	600	1550

Note

Number of nest box controls refers to the summed number of visits at all nest boxes.

## 4 DISCUSSION

### 4.1 Technical comments

All trail cameras set to determine the time of the first egg laid and hatched worked without batteries replacement, while the SD memory card was changed two to three times. Despite the high number of photos recorded for each nest, browsing them to find the eggs laid or hatched took usually <5 min and was performed in the field during nest box control. Aside from lacking the option of switching off the PIR motion sensor and too intensive IR LED flash, which was easy to fix (see Methods, Figure 1), we recorded just a few drawbacks of the system. One was related to the focal length of the camera. Although 250 mm lens enabled focusing at a minimal distance of 25 cm, in some cases the height of nests exceeded 20 cm (Podkowa & Surmacki, 2017), which markedly shortened the distance between the nest content and the camera lens. In such cases, images had a narrow frame and were slightly out of focus (Figure 3). Nevertheless, the frame was usually wide enough to cover the entire nest cup and the image in the photo was sufficiently legible to detect the presence of eggs or the evidence of hatching. One drawback of the model of trail cameras that we used is that they could not be powered by rechargeable batteries due to their low cell voltage which is usually 1.2 V. Occasionally (~10% of cameras), self‐acting changes of the date and clock occurred.

It is important to stress that all of the above drawbacks could be eliminated by replacing Bushnell Natureview^®^ HD with another model of a trail camera. For example, the S8080 trail camera has a built‐in PIR motion sensor switch, 100° lens, 12 cm minimal focusing distance and smooth IR LED control. As a result, it provides clear images of the entire nest box (Video S2). Additionally, it could be powered by rechargeable batteries and has a built‐in LCD color monitor for previewing photos and videos. Finally, it has smaller dimensions (13 × 10 × 7 cm) thus decreasing the box dimensions needed to fit the camera. It is also important to note that trail camera hardware could be modified to meet customers’ needs (e.g., Littlewood et al., 2021; Uhe et al., 2020; Welbourne et al., 2019).

Overall, the use of trail cameras for nest box monitoring has several advantages when compared to other equipments used for nest box monitoring. First, using trail cameras is relatively inexpensive: Bushnell Natureview^®^ HD retails for ~250 Euro, however, most trail models can be purchased for less than 150 Euro. Second, trail cameras could be easily swapped between boxes, which is especially important in studies with high numbers of boxes involved and/or when box occupation pattern changes dynamically. Finally, trail cameras could be easily hidden inside the nest box with no additional equipment left visible outside (compare to Hereward et al., 2021).

### 4.2 Comparison of TCM to other methods

The theoretical calculations showed that TCM is more efficient and precise when compared to traditional field NBC. Although average daily time expenditures are comparable for both methods, the total time expenditures in NBC are about 75% higher than in TCM. Such a difference is caused by the fact that in order to establish the hour of egg laying and hatching each nest box has to be controlled three times a day for 3–5 consecutive days. In a consequence, NBC requires daily visits in a field. In TCM, on the other hand, visits are needed mainly to install and retrieve trail cameras. Hence, the number of field working days in NBC is over three times higher in the case of the hour of egg laying survey, and even more than six times higher in the case of egg hatching survey.

Despite the huge time expenditures, the accuracy of the data collected with NCB is significantly lower than in TCM. The errors for the hour of egg laying in NCB and TCM are on average ±60 and ±14.5 min, respectively. Even greater disproportions were noted for the errors in the hour of egg hatching. In the NBC, depending on the time period, the error was ±3–5.5 h, while in TCM it was about ±0.5 min. Taking the above into account, the NBC method may be considered as unsuitable for measuring the variance of hours of eggs laying and hatching.

One weak point of the TCM in determining the egg laying time is that some of the eggs (15% in this study) could be missed by the camera, because the female may cover it with the nest lining immediately after laying. Nevertheless, the obtained sample size is still high enough to describe the daily hourly pattern egg laying, which remains unknown for most bird species. An alternative for TCM is continuous video recording of the nest box interior (e.g., Haftorn, 1996; Houdelier et al., 2007). This method was used to determine the time of eggs laying based on the characteristic behavior of the females (Haftorn, 1996; Houdelier et al., 2007). The downside of this method is the large number of recordings that need to be analyzed and this is probably why it has been used so far on a small number of nest boxes (for example, 9 nests in Haftorn, 1996 and 11 nests in Houdelier et al., 2007). To date, there was no study which aimed to determine the daily pattern of egg hatching by means of automated photography or video recordings.

The fact that birds hatch 24 h a day means that the hour of hatching can have a significant impact on the assessment of the date of hatching, which is vital for calculating the nestlings’ age. In a traditional approach, the hatching date is determined by daily nest checks, usually performed at the same time of the day (Nilsson & Svensson, 1993; Visser et al., 1998, but see Winkel (1970) for the alternative method). Assuming that field nest box check takes place between 5:00 and 6:00 p.m., according to our findings, 8%–15% clutches hatched after this time. It means that these hatchlings will be detected on the next day and their age will be underestimated by one day. The rate of erroneously aged nestling could be higher, if the field nest check is performed earlier, e.g., between 2:00 and 3:00 p.m. (18%–33% broods with misestimated age).

The high variance in the hour of hatching may have significant consequences for the parameters of nestlings. We showed that the mean brood’s mass in the second day of life positively correlates with the daytime the first hatchling spent in the nest on the day of hatching. The most obvious explanation of this result is that nestlings that hatched earlier in the day received more food from their parents compared to nestlings that hatched later. This relationship was not significant on the 12th day of life; however, it is important to remember that our methodological approach was very rough, because it did not take into account between‐nestling differences in the hatching time. Further research is needed to determine if other aspects of nestlings’ health and development depend on the time of day they hatch.

### 4.3 Daily patterns of egg laying and hatching

The hourly pattern of egg laying that we documented in Great Tits is consistent with earlier findings based on smaller sample sizes (Haftorn, 1996). Females laid eggs in a relatively short time range (ca. 2 h) around sunrise. Compared to data gathered by Haftorn (1996), birds from our population laid eggs on average 24 min earlier (in relation to the sunrise) and within a shorter time range (127 min compering to 150 min). There are several possible reasons for these differences. First, Haftorn (1996) collected data between May and June, while our dataset was confined to April. Second, our finding was based on a larger sample size of nests (56 compared to 9). Third, the population studied by Haftorn (1996) was located farther North compared to ours. Laying eggs early in the morning is observed in many, especially small species, which may be beneficial for several reasons (e.g., Feare et al., 1982, reviewed in McMaster et al., 2004). For example, it may reduce the probability of egg breakage in oviducts during morning activities and/or decrease body mass after egg laying which in turn may increase the efficiency of foraging (McMaster et al., 2004). Some authors suggest that egg‐laying time is tuned to the best fertilization time window, which in general is possible after the previous egg is laid (Birkhead, 1988; Weatherhead et al., 1991). All the above hypotheses are plausible in the case of Great Tit.

The timing of egg hatching has never been studied in Great Tit, and information for other species is very scarce. Skutch (1952) determined the timing of egg hatching in 11 neotropical open‐nesters by assigning it to three wide and uneven time intervals (dawn – noon, noon – nightfall, night). According to Skutch (1952), there are two timing strategies of hatching: random and non‐random. The pattern of hatching in Great Tit that emerged from our study cannot be easily classified. On the one hand, hatchings were recorded over the entire 24‐h day. On the other hand, two peaks in hatching could be distinguished: one around the sunrise and another in the afternoon. The mechanisms responsible for the observed schedule of hatching in the Great Tit remain speculative. Hatch timing could be caused by the embryo’s daily rhythm or/and the time of onset of incubation Skutch (1952). Moreover, bird embryos may sense the environment outside of the eggshell, e.g., temperature, humidity, light, sound, or scent (Caspers et al., 2017; Mariette et al., 2018; Maurer et al., 2011; Noguera & Velando, 2019; Rumpf & Tzschentke, 2010), and wait for the most favorable moment to emerge. Potential factors determining the variation of hatching hour and its functional significance open new research avenues for avian embryonic development.

### 4.4 Application of the trail camera for other tasks and animal species

The presented method can be applied for a variety of purposes and animal taxa. The continuous motion‐triggered video data could be used to survey the frequency and duration of activities like nest building, offspring provisioning (Podkowa et al., 2019), nest sanitation, and nest box occupancy by roosting animals (Video S1, Video S3, Video S4). Time‐lapse photos can be used to monitor incubation attentiveness or to determine the fledging time in birds (Figure S1). Time‐lapse recordings would be also appropriate in studies on insects and other invertebrates. TCM is suitable to study other cavity‐nesting taxa such as mammals, reptiles, and amphibians, which use boxes for breeding, hibernation, roosting, or temporary shelter. Depending on the goal of the research and the study species, nest boxes could be modified to fix trail camera to the ceiling, floor, or sidewall, or to use more than one camera if necessary.

## CONFLICT OF INTEREST

The authors involved in the preparation of this manuscript have no conflicts of interest to declare.

## AUTHOR CONTRIBUTIONS

**Adrian Surmacki**: Conceptualization (lead); data curation (equal); formal analysis (lead); funding acquisition (lead); investigation (equal); methodology (lead); project administration (lead); supervision (lead); visualization (lead); writing‐original draft (lead); writing‐review & editing (lead). **Paweł Podkowa**: Data curation (equal); formal analysis (supporting); investigation (equal); methodology (supporting); writing‐original draft (supporting); writing‐review & editing (supporting).

## Supporting information

Fig S1

Video S1

Video S2

Video S3

Video S4

Appendix S1

Supplementary Material

## Data Availability

Data for the statistical analyses can be found at Dryad entry https://doi.org/10.5061/dryad.fj6q573wh.
