# Supplementary material for: The use of trail cameras to monitor species inhabiting artificial nest boxes
Source: Ecol Evol. 2022 Feb 7;12(2):e8550. doi: 10.1002/ece3.8550 (PMC8820111; doi:10.1002/ece3.8550)

Figure S1. Examples of application of sequentially recorded photos within a given time lapse ("Field Scan" mode in Bushnell trail camera) in studying birds' behaviour. The first three rows (color photos): nest attendance during incubation; the last row (black and white photos): timing of fledging.

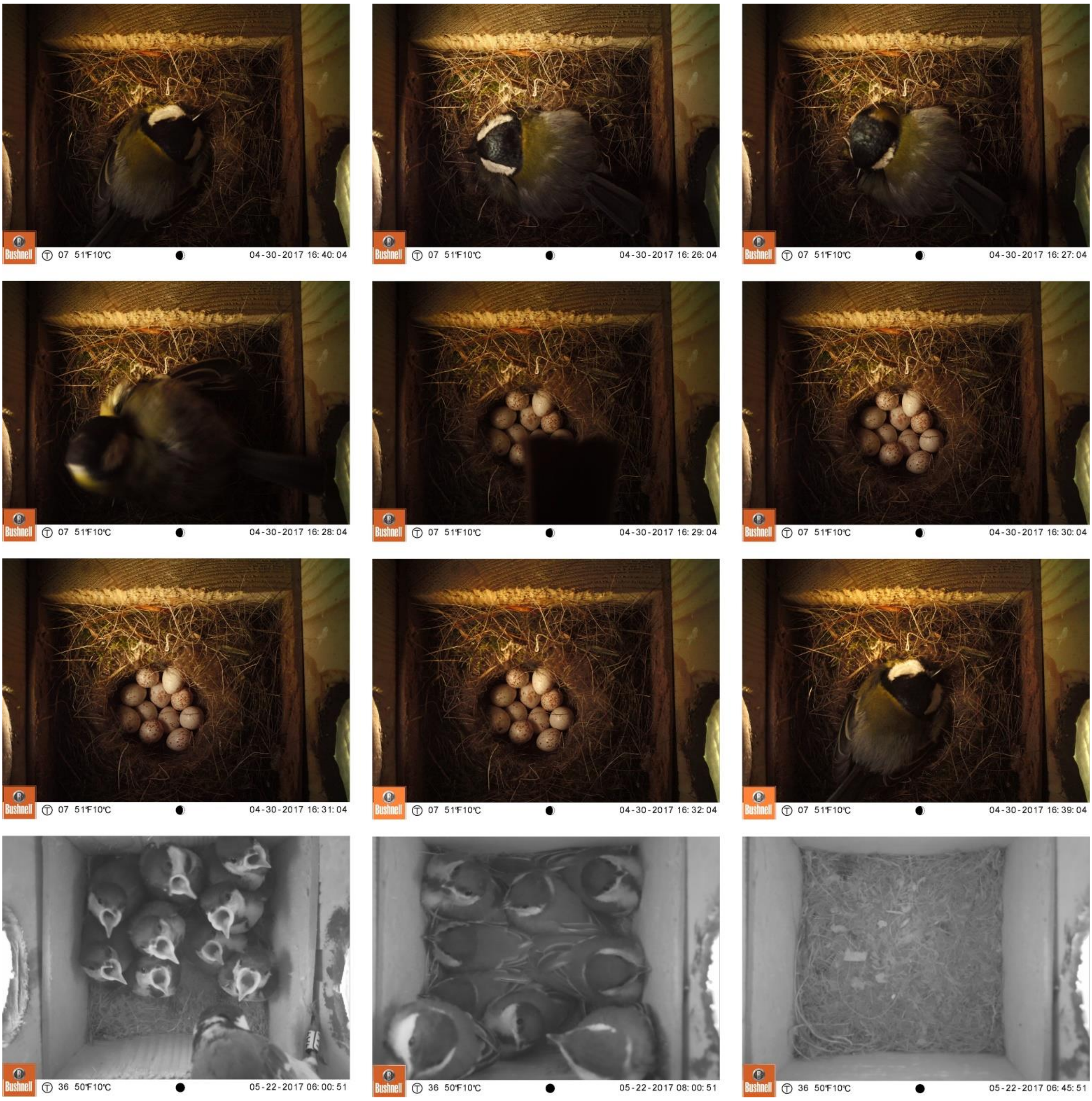

Supplement: Supplementary file 1 — Fig S1 [file ECE3-12-e8550-s004.pdf]
