# Supplementary material for: The use of trail cameras to monitor species inhabiting artificial nest boxes
Source: Ecol Evol. 2022 Feb 7;12(2):e8550. doi: 10.1002/ece3.8550 (PMC8820111; doi:10.1002/ece3.8550)
Supplement: Supplementary file 6 — Appendix S1 [file ECE3-12-e8550-s005.pdf]

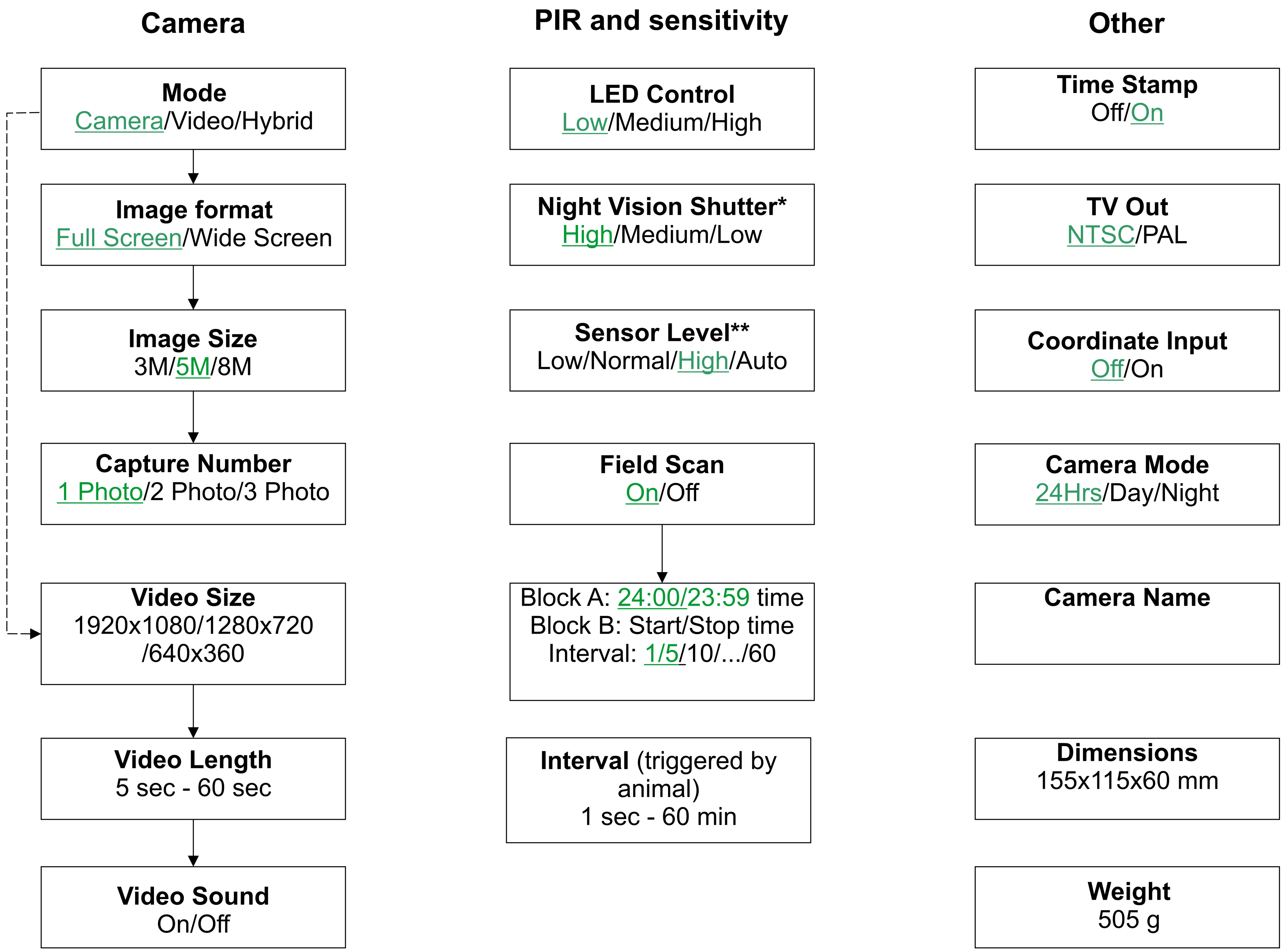

*Night Vision Shutter - „High” means that shorter exposition freezes motion better at the expense of brightness

**Sensor Level - „High” enables to detect even small differences in temperature between the bird’s body and the surrounding area
