# Supplementary material for: The use of trail cameras to monitor species inhabiting artificial nest boxes
Source: Ecol Evol. 2022 Feb 7;12(2):e8550. doi: 10.1002/ece3.8550 (PMC8820111; doi:10.1002/ece3.8550)
Supplement: Supplementary file 7 — Supplementary Material [file ECE3-12-e8550-s001.docx]

**Description of Supporting Information**

| **Filename** | **Description** |  |
| --- | --- | --- |
| Appendix 1_SuppInfo.pdf | **Appendix S1**. Parameters and settings of Bushnell trail camera used in the study | |
| Figure_S1_SuppInfo.pdf | **Figure S1.** Examples of applications of sequentially recorded photos within a given time lapse (“Field Scan” mode in Bushnell trail camera) in studying birds' behavior. The first three rows (color photos): nest attendance during incubation; the last row (black and white photos): timing of fledging |  |
| Video_S1_SuppInfo.AVI  AVI video, 4.8 MB | **Video S1.** An original video from a Bushnell trail camera recorded in IR light shows nestling feeding and feces removal. |  |
| Video_S2_SuppInfo.AVI  AVI video, 3.7 MB | **Video S2**. An original video from a Bushnell trail camera recorded in daylight that shows nestling feeding. |  |
| Video_S3_SuppInfo.MP4  MPEG-4 video, 9,9 MB | **Video S3**. An original video from TV5140 trail camera recorded in IR light which shows nestling feeding. The adult bird seems to be aware of camera presence. Note the wider frame compared to the Bushnell videos. |  |
| Video_S4_SuppInfo.MPp4  MPEG-4 video, 10,5 MB | **Video S4**. An original video from the Bushnell trail camera recorded in IR light that shows a Nuthatch during night roost in the Autumn. |  |
